# Patient safety attitudes of frontline healthcare workers in Lahore: A multicenter study

**DOI:** 10.12669/pjms.38.1.4964

**Published:** 2022

**Authors:** Javed Arkam, Shehnoor Azhar, Khalid Saeed Khan, Arifa Aman

**Affiliations:** 1 Prof. Javed Akram, MBBS, FRCP. Vice Chancellor, University of Health Sciences, Khayabaan e Jamia Punjab, Lahore 54000, Pakistan; 2 Dr. Shehnoor Azhar, BDS, MPH. Assistant Professor, Department of Public Health, Doctoral Candidate Public Health, University of Granada, Spain. University of Health Sciences, Khayabaan e Jamia Punjab, Lahore 54000, Pakistan. Distinguished Investigator, Department of Clinical Medicine & Public Health, University of Granada, Spain; 3 Prof. Khalid Saeed Khan, FCPS, MRCGOB. Distinguished Investigator, Department of Clinical Medicine & Public Health, University of Granada, Spain; 4 Dr. Arifa Aman, MBBS. Private Medical Practitioner, Lahore, Pakistan

**Keywords:** Communications, Culture, Frontline, Hospitals, Patient safety

## Abstract

**Objectives:**

To evaluate patient safety attitudes of the frontline health workers in hospitals of Lahore, Pakistan.

**Methods:**

A self-administered Safety Attitudes Questionnaire (SAQ) survey was deployed in five hospitals across Lahore, Pakistan (July 2019 to June 2020). A total of 1250 consecutive consenting nurses and postgraduate trainee physicians of under five years working experience were recruited. Assessment for each of the six subdomains (teamwork climate, safety climate, job satisfaction, stress recognition, perception of management, working conditions) was done on a 0-100 scale. Multivariate analyses examined their relationship with job cadre (nurses and physicians), duration of respondents’ work experience (< 2 years, 3 - 4 years, > 4 years), and hospital sector (private and public).

**Results:**

The response rate was 97% (1212 individuals; 765 nurses, 447 physicians). Nurses scored less than physicians in teamwork climate (-2.4, 95% CI -4.5 – -0.2, p=0.02) and stress recognition (-10.6, 95% CI -13.5 – -7.7, p<0.001), but more in perception of management (4.2, 95% CI 1.5 – 6.8, p=0.002) and working conditions (3.4, 95% CI 0.66 – 6.2, p=0.01). Increasing work experience was related to greater scores in all subdomains. Private hospitals scored generally higher than public ones.

**Conclusion:**

Duration of job experience was positively correlated with patient safety attitudes of hospital staff. These finding could serve as the baseline to shape staff perceptions by cadre in both public and private sector hospitals.

## INTRODUCTION

Medical errors are the third leading cause of death in United States after cardiovascular conditions and cancers.^1^ Over 150,000 lives are annually lost to preventable adverse events rooted in misdiagnosis, poorly skilled workforce, and communication breakdowns in hospitals. Their high frequency reported elsewhere make them a global problem.^2^,^3^ In Europe, direct costs of hospital-acquired infections range from 37,000 to 110,000 deaths/year besides financial burden of €5.4 billion/year.^1^,^4^,^5^ Hospitalization-associated adverse events and infections in low- and middle-income countries tend to be 20 – 50% higher than those in high-income countries, making patient safety an even bigger issue for developing countries.^6^-^8^

Preventing harm and improving patient safety require a care quality framework care quality framework aimed at professionalizing the workforce continuously through targeted strategies.^9^ Among several tools and metrics that exist to assess patient safety attitudes, Safety Attitudes Questionnaire (SAQ) has well-established cross-cultural strength, superior psychometric properties, and validity for inter-hospital comparisons.^10^-^12^ Previous studies have been unreliable due to smaller sample sizes and being limited to a hospital department or a workforce cadre.^3^,^6^,^13^ In addition, there remains concerns for generalizability of reported findings in absence of representation from both public and private sector hospitals. Without a reliable baseline that reflects the scope of the problem, there’s limited attention being paid to the patient safety culture hence draining precious resources.^14^ But the issue comes to the fore during unforeseen clinical scenarios such as COVID-19 pandemic. We formally evaluated staff attitudes in a multicenter study involving five tertiary care hospitals to examine factors underpinning hospital patient safety culture.

## METHODS

The questionnaire survey was conducted and reported according to published methodological guidance.^15^ It was approved by Ethical Review Committee (Reference no UHS/REC/1732) at University of Health Sciences Lahore, and all participants provided informed consent. A self-administered study tool was deployed among consenting hospitals across Lahore, Pakistan (July 2019 to June 2020). Invitations were sent to Principals of eight affiliated teaching hospitals of which five agreed to participate. Of these, two were private and three were in the public sector.

### Questionnaire and piloting:

All respondents provided individual informed consent. Prior permission for use of generic short-form Safety Attitudes Questionnaire (SAQ 2006) was obtained from its authors via email. It was chosen for well-established cross-cultural strength, superior psychometric properties, and validity for inter-hospital comparisons. To be recorded on Likert-like scale, it comprised 31 response items unequally divided into six subdomains namely teamwork climate, safety climate, job satisfaction, stress recognition, perception of hospital management, and working conditions. The remaining five items did not belong to any subdomain. Each response item was scored individually (Strongly Disagree = 0, Slightly Disagree = 25, Neutral = 50, Slightly Agree = 75, Strongly Agree = 100) to compute an aggregate subdomain score on the scale of 0 to 100. The response option “Not Applicable” was not used considering the range of responses given by the pilot sample. Three negatively worded items were reverse scored. The original English version of questionnaire was pilot tested on 43 clinical staff to establish survey procedures, to build a bespoke data management system, and to obtain relevant information for sample size estimation. Internal consistency of the piloted sample was Cronbach α 0.93.

### Power and sample size:

The variances obtained from the pilot data were deployed in *a priori* sample size calculations with power or 1 – β of 80% and α 0.05.^16^ The nurse to physician ratio was set at 2:1 proportionate to staff distribution in participating hospitals. To obtain a representative sample we wanted to enroll at least 10% of the estimated 12,500 frontline staff in participating hospitals. This required inflation of the calculated sample size by 25%. Thus, in total 1250 participants were invited. This sample size allowed for a proportion of non-response and permitted powerful multivariate analysis.

### Sample and data collection:

We invited frontline clinical staff divided into two groups: nurses, and postgraduate trainee physicians, with no more than five years of work experience at the same hospital. Four considerations informed the choice of study sample. Firstly, frontline hospital staff are the patients’ first point of contact with any hospital. Second, they are the staff most frequently in contact with the patient during a typical hospitalization episode. Third, they comprise majority of the hospital workforce. Finally, assessment of attitudes early in the career better professional development strategies. Clinical staff (medical faculty, hospital physicians, paramedical staff, technologists) with service tenures greater than five years or those with history of employment at other hospitals were excluded.

A data collection plan was devised for hospitals in consultation with respective administrations but not disclosed to participating departments namely medicine, surgery, gynecology and obstetrics, pediatric, and central accident and emergency. It expected to minimize desirability bias that might have arisen out of pre-notified visits. Before distributing the questionnaires at study sites, eligible staff were informed of study objectives including the requirement to maintain data confidentially for up to 10 years. Duly filled out questionnaires were required to be returned within three working days. Data were collected during working shifts (morning, evening, night) on weekdays in a six months period starting July 2019.

### Data Analysis:

Data were analyzed in STATA 14 (College Station, TX) as per planned analytical framework. Demographic characteristics were to be tabulated. Multivariate regression adjusted for differences in hospitals to determine the relationships between each of the six subdomains as outcome and independent variables – job cadre, duration of experience, and public or private sector institutions. Regression results were summarized as coefficients, 95% Confidence Interval (CI), and p-values significant at level of 0.05 for rejection of null hypotheses.

## RESULTS

The overall response rate was 97%. Of the total 1212 study participants, 76% were females. About 47.4% were recruited within last two years, 27.7% between two years to four years while 24.8% had been working for more than four years. Participants scored highest for job satisfaction (64.3 ±22.2) as summarized in Table-I. Both study groups (physicians 59.3 ±17.8 and nurses 59.3 ±17.8) scored nearly equal for teamwork climate. Physicians (65.7 ± 24.5) scored higher in stress recognition than nurses (57 ±23.1). For remaining four sub-domains, nurses scored higher. Across hospitals there were variations in that private sector scored higher than public.

**Table I:** Safety attitudes sub-domain scores reported by clinical staff working in five hospitals in Lahore, Pakistan (Mean scores with standard deviations (SD) scaled 1 to 100). Hospitals abbreviated – LGH, JHL, SIMS in public sector with GTTH, SMCH in private.

	LGH Mean ±SD		JHL Mean ±SD		SIMS Mean ±SD		GTTH Mean ±SD		SMCH Mean ±SD		All Hospitals Mean ±SD	

Sub-domains	Total N = 368		Total N = 392		Total N = 324		Total N = 77		Total N = 51		Total N = 1212	

	Physician n = 104	Nurse n = 264	Physician n = 142	Nurse n = 250	Physician n = 141	Nurse n = 183	Physician n = 22	Nurse n = 55	Physician n = 38	Nurse n = 13	Physician n = 447	Nurse n = 765
Teamwork climate	64.0 ±16.0		54.4 ±19.7		56.5 ±15.9		66.6 ±16.4		64.3 ±19.3		59.1 ±18.0	
	62 ±16.4	65 ±15.9	56.2 ±19.1	53.5 ±20.0	56.5 ±16.0	56.5 ±15.9	64.5 ±16.3	67.4 ±16.6	70.1 ±18.8	47.4 ±7.3	59.3 ±17.8	59.1 ±18.2
Safety climate	63.4 ±14.6		53.6 ±18.8		55.6 ±12.3		65.7 ±15		61.9 ±17.4		58.2 ±16.3	
	61.3 ±12.7	64.3 ±15.2	52.3 ±20.4	54.4 ±17.9	53.7 ±12.4	57.0 ±12.0	64.6 ±15.9	66.2 ±41.7	67.5 ±15	45.3 ±13.0	56.7 ±16.5	59.1 ±16.2
Job satisfaction	71.1 ±19.8		58.8 ±25.3		60.2 ±18.1		72.6 ±21.6		71.3 ±18.8		64.3 ±22.2	
	65.2 ±20.4	73.5 ±19.2	62 ±25.4	57.1 ±25.1	57.3 ±19.9	62.4 ±16.4	66.5 ±19.1	75.0 ±22.2	75.9 ±17.4	58.0 ±16.8	62.7 ±22.2	65.3 ±22.1
Stress recognition	63.2 ±20.5		59.6 ±27.8		58.6 ±20.1		47.4 ±28.3		72.5 ±22.7		60.2 ±24.0	
	64.3 ±23.5	62.8 ±19.2	71.4 ±26.4	53 ±26.4	59.9 ±22.1	57.6 ±18.4	61.3 ±25.1	41.8 ±27.7	72.2 ±22.8	71.6 ±23.1	65.7 ±24.5	57 ±23.1
Perceptions of management	59.9 ±22.4		49.8 ±23.5		54.8 ±16.1		59.8 ±21.1		57.4 ±23.9		55.1 ±21.7	
	48.7 ±27.1	64.3 ±18.6	50.1 ±23.5	49.7 ±23.6	51.5 ±14.6	57.3 ±16.8	56.3 ±20.2	61.1± 21.5	60 ±24.6	50 ±20.8	51.3 ±22.1	57.4 ±21.1
Working conditions	66.0 ±20.6		52.8 ±25.4		53.6 ±20.7		65.0 ±24.4		65.8 ±23.2		58.3 ±23.4	
	56.6 ±23.2	69.6 ±18.2	52.7 ±27.5	52.9 ±24.3	50.6 ±21.2	55.9 ±20.1	59.3 ±24.5	67.2± 24.2	66.9 ±22.5	62.5 ±25.9	54.5 ±24.4	60.6 ±2.6

***Abbreviations:*** LGH Lahore General Hospital, JHL Jinnah Hospital Lahore, SIMS Services Institute of Medical Sciences, GTTH Ghurki Teaching Trust Hospital, SMCH Sharif Medical City Hospital.

The multivariate regression models (Table-II) showed lower nurse’ scores than physicians’ in teamwork climate (-2.4, 95% CI -4.5 – -0.2, p=0.02) and stress recognition (-10.6, 95% CI -13.5 – -7.7, p<0.001), but higher in perception of management (4.2, 95% CI 1.5 – 6.8, p=0.002) and working conditions (3.4, 95% CI 0.66 – 6.2, p=0.01). Increasing work experience was related to greater scores in teamwork climate (3.4, 95% CI 2.1 – 4.6, p<0.001), safety climate (3.1, 95% CI 2.0 – 4.2; p<0.001), job satisfaction (4.5, 95% CI 3.0 – 6.0, p<0.001), stress recognition (2.5, 95% CI 0.8 – 4.2, p=0.003), perception of management (3.2, 95% CI 1.6 – 4.7, p<0.001), and working conditions (3.5, 95% CI 1.9 – 5.1, p<0.001). Private hospitals scored higher than public in teamwork climate (17.1, 95% CI 12.7 – 21.6, p<0.001), safety climate (16.5, 95% CI 12.4 – 20.5, p<0.001), job satisfaction (22.1, 95% CI 16.6 – 27.5, p<0.001), perception of management (10.3, 95% CI 4.8 – 15.7; p<0.001), and working conditions (22.1, 95% CI 16.3 – 27.9, p<0.001).

**Table II:** Relationship of participants’ characteristics with patient safety sub-domains scores using linear regression analysis.

Sub-domains						

	Teamwork climate	Safety climate	Job satisfaction	Stress recognition	Perceptions of management	Working conditions
***Univariate***						
Cadre (Nurses vs Physicians)	-0.1	2.3	2.64	-8.7	6.0	6.0
CI	-2.2 – 1.9	0.4 – 4.2	0.05 – 5.2	-11.5 – -5.9	3.5 – 8.5	3.3 – 8.8
p	0.8	0.01	0.04	0.001	0.001	0.001
Experience (years)	2.9	3.12	4.4	0.8	3.8	4
CI	1.7 – 4.2	2.01 – 4.2	2.8 – 5.9	-0.8 – 2.4	2.3 – 5.3	2.4 – 5.6
P	0.001	0.001	0.001	0.3	0.001	0.001
Sector (Private vs Public)	7.2	6.6	8.6	-3.1	4.1	7.7
CI	4.0 – 0.5	3.6 – 9.6	4.64 – 12.7	-7.5 – 1.2	0.1 – 8	3.4 – 12
P	0.001	0.001	0.001	0.1	0.04	0.001
**Multivariate**						
Cadre (Nurses vs Physicians)	- 2.4	0.2	-0.3	-10.6	4.2	3.4
CI	-4.5 – -0.2	-1.6 – 2.19	-3 – 2.2	-13.5 -- -7.7	1.5 – 6.8	0.66 – 6.2
P	0.02	0.7	0.7	0.001	0.002	0.01
Experience (years)	3.4	3.1	4.5	2.5	3.2	3.5
CI	2.1 – 4.6	2.0 – 4.2	3.00 – 6.0	0.8 – 4.2	1.6 – 4.7	1.9 – 5.1
P	0.001	0.001	0.001	0.003	0.001	0.001
Sector (Private vs Public)	17.1	16.5	22.1	0.9	10.3	22.1
CI	12.7 – 21.6	12.4 – 20.5	16.6 – 27.5	-5 – 6.9	4.8 – 15.7	16.3 – 27.9
p	0.001	0.001	0.001	0.7	0.001	0.001

Data presented as coefficient, 95% Confidence Interval (CI), and p-values.

## DISCUSSION

This multicenter study involving five tertiary care hospitals showed that physicians, compared to nurses, had more positive perceptions in relation to teamwork climate and stress recognition. However, nurses had comparatively more positive perceptions of management and working conditions. Overall, increasing duration of work experience correlated positively with all patient safety attitudes. Logistic regression in Table-II shows that private sector hospitals had comparatively more positive perceptions across all areas of attitudes except stress recognition.

This study has several strengths. It deployed a questionnaire with well-established psychometric properties in its original form after validating locally in a pilot study, completed the study without significant participants loss while meeting the *a priori* power and sample size estimates, and performed multivariate analyses adjusting for confounders. It included staff from major hospitals in both public and private sectors, captured around a tenth of the workforce in a multicenter multispecialty setting, proportionately sought representation of nursing and physician cadres, and administered the survey across all three work shifts. Our findings are trustworthy and generalizable. Future scholarship can build further upon these findings by gauging the progress against comparisons with high-income countries as summarized in Table-III.^1^

**Table III:** Comparison of SAQ scores (Mean ± SD) of respondents with global standards (Scale 1 - 5).

Items	Turkey M (SD)	Global Standard M (SD)	Denmark M (SD)	Switzerland M (SD)	Pakistan this study M (SD)
***Teamwork climate***					
Nurse input is well-received in this clinical area	3.7 (1.1)	3.9 (1.05)	4.2 (0.9)	4.4 (0.73)	3.2 (1.3)
Difficult to speak up when problem with patient care*	3.3 (1.4)	2.4 (1.21)	3.9 (1.2)	4.3 (0.98)	3.0 (1.2)
Disagreements area are resolved appropriately	3.3 (1.3)	3.5 (1.10)	3.7 (1.2)	4.0 (0.85)	3.2 (1.2)
I have the requisite support for care for patients	3.5 (1.1)	3.9 (0.99)	4.3 (0.8)	4.0 (0.82)	3.5 (1.1)
If something not understood, it’s easy to ask questions	3.8 (1.0)	4.1 (0.96)	4.5 (0.8)	4.5 (0.72)	3.6 (1.1)
Physicians and nurses work as well-coordinated team	3.5 (1.1)	3.7 (1.07)	3.9 (1.0)	3.6 (0.78)	3.5 (1.2)
***Safety climate***					
I would feel safe being treated here as a patient	3.6 (1.2)	4.0 (1.0)	4.0 (1.0)	3.9 (0.7)	3.3 (1.2)
Medical errors handled appropriately in this clinical area	3.8 (1.1)	3.4 (1.0)	4.0 (1.0)	4.0 (0.7)	3.4 (1.2)
I know the channels for queries regarding patient safety	3.1 (1.3)	3.8 (1.0)	4.0 (1.0)	4.4 (0.7)	3.4 (1.1)
I receive appropriate feedback about my performance	2.4 (1.3)	3.2 (1.2)	3.4 (1.2)	3.7 (0.9)	3.3 (1.2)
In this clinical area, it is difficult to discuss errors*	3.1 (1.3)	2.5 (1.1)	4.0 (1.1)	4.0 (0.9)	2.7 (1.2)
Encouraged by peers to report patient safety concerns	3.3 (1.2)	4.0 (0.9)	3.6 (1.1)	3.8 (0.9)	3.5 (1.2)
Culture here makes it easy to learn from errors of others	3.2 (1.2)	3.9 (1.0)	3.6 (1.1)	3.9 (0.9)	3.5 (1.1)
***Job security***					
I like my job	4.3 (0.8)	4.3 (0.8)	4.5 (0.8)	4.6 (0.6)	3.7 (1.2)
Working here is like being part of a large family	3.0 (1.4)	3.1 (1.3)	3.4 (1.1)	3.6 (0.9)	3.5 (1.1)
This is a good place to work	3.0 (1.3)	3.7 (1.0)	4.1 (0.9)	4.2 (0.7)	3.5 (1.1)
I am proud to work in this clinical area	3.1 (1.3)	3.7 (1.0)	4.1 (0.9)	4.1 (0.8)	3.4 (1.2)
Morale in this clinical area is high	3.2 (1.3)	2.9 (1.2)	4.1 (0.9)	4.0 (0.7)	3.4 (1.2)
***Stress recognition***					
Excessive workload impairs my performance	4.0 (1.2)	3.8 (1.1)	4.1 (1.1)	3.4 (1.1)	3.5 (1.2)
I am less effective at work when fatigued	3.9 (1.2)	3.9 (1.0)	4.0 (1.0)	3.6 (1.1)	3.4 (1.2)
I am more likely to make errors in tense situations	3.5 (1.4)	3.7 (1.1)	3.6 (2.0)	3.6 (1.1)	3.4 (1.2)
Fatigue impairs me during emergency situations	3.8 (1.2)	3.0 (1.2)	3.3 (1.3)	-	3.2 (1.3)
***Perception of management***					
Management supports my daily efforts	2.4 (1.3)	2.7 (1.1)	3.9 (1.0)	2.8 (1.1)	3.1 (1.2)
Management don’t knowingly compromise pt safety	3.9 (1.2)	3.2 (1.2)	4.0 (1.1)	3.3 (1.4)	3.1 (1.2)
Management is doing a good job	-	-	4.0 (1.1)	-	3.2 (1.2)
Problem personnel are dealt with constructively by Mgt	2.5 (1.3)	2.8 (1.1)	3.7 (1.1)	3.3 (0.9)	3.1 (1.1)
I get adequate, timely info about events from Mgt	2.7 (1.3)	3.1 (1.0)	3.6 (1.1)	3.6 (0.9)	3.2 (1.1)
***Working conditions***					
Staffing in this clinical area is enough to handle pts	2.2 (1.4)	2.6 (1.3)	3.0 (1.3)	3.4 (1.0)	3.1 (1.3)
This hospital does a good job of training new personnel	3.2 (1.3)	3.5 (1.1)	3.9 (1.2)	4.0 (0.9)	3.3 (1.2)
All necessary information is routinely available to me	3.5 (1.1)	3.5 (1.0)	4.2 (0.9)	4.1 (0.7)	3.4 (1.2)
Trainees in my discipline are adequately supervised	3.3 (1.2)	3.5 (1.1)	3.7 (1.1)	4.2 (0.8)	3.4 (1.2)

- Denotes missing values.

### Limitations of the study:

It focused on frontline clinical staff who are most frequently in contact with patients. Thus, our findings concerning attitudes included in Tables I and II may not be considered generalizable to other hospital workforce cadres that include technologists, senior faculty, and administrators. However, these groups are not as directly involved in service provision. Predominance of females in the study sample is likely linked to nursing profession. The survey design is limited in its ability to capture variations in attitudes that are sensitive to daily events and clinical scenarios.^15^,^17^ A longitudinal design was more appropriate to account for such variations at a dynamic workplace like a hospital. Our study provides the foundations on which to build longitudinal evidence.

Existing literature links variations in patient safety attitudes with geography both within and outside. In few studies reported from Pakistan on this topic, clinical staff working in Karachi had negative attitudes compared to those in Lahore.^18^,^19^ It warrants more research on sociocultural context related to poor treatment outcomes since little is taught on the subject during undergraduate training.^20^,^21^ Patient safety attitudes have implications for health services delivery in COVID-19 era and beyond.^22^ Our results could be the baseline for local hospital administrators to project a safety climate recently put under strain due to perceived vulnerability of healthcare workers while at work during the ongoing pandemic.

## CONCLUSION

Duration of work experience correlated with all subdomains of patient safety, unlike staff cadre and hospital sectors that correlated with specific aspects. Promotion of positive patient safety attitudes needs underpinning data to direct development of targeted strategies so that care quality and treatment outcomes could be improved.
